# X-Ray snapshots of a pyridoxal enzyme: a catalytic mechanism involving concerted [1,5]-hydrogen sigmatropy in methionine γ-lyase

**DOI:** 10.1038/s41598-017-05032-6

**Published:** 2017-07-07

**Authors:** Dan Sato, Tomoo Shiba, Tsuyoshi Karaki, Wataru Yamagata, Tomoyoshi Nozaki, Takashi Nakazawa, Shigeharu Harada

**Affiliations:** 1 Graduate School of Science and Technology, Department of Applied Biology, Kyoto Institute of Technology, Sakyo-ku, Kyoto, 606-8585 Japan; 2 Department of Parasitology, National Institute of Infectious Diseases, 1-23-1 Toyama, Shinjuku-ku, Tokyo, 162-8640 Japan; 3 Department of Chemistry, Nara Women’s University, Nara, 630-8506 Japan

## Abstract

Pyridoxal 5′-phosphate (PLP)-enzymes are essentially involved in amino acid and amine metabolism of a wide variety of organisms. Despite their extensive biochemical studies, there are little evidence and structural data to comprehensively elaborate the catalytic mechanism. We obtained X-ray snapshots of l-methionine γ-lyase from *Entamoeba histolytica* (EhMGL), a PLP-enzyme catalyzing the γ-elimination reaction of methionine. Here, we suggest a catalytic mechanism of EhMGL by using the X-ray snapshots covering all stages of this multistep catalysis reaction. Initial formation of a Michaelis complex is followed by the migration of double bond from the C4′=Nα–Cα moiety in an intermediate PLP-methionine imine to C4′–Nα=Cα in pyridoxamine 5′-phosphate (PMP)-α,β-dehydromethionine imine without intervention of a putative quinonoid intermediate. The enzyme can facilitate the subsequent γ-elimination of methanethiol by the possible general acid-base catalysis of Tyr108 for the E1cB mechanism, enabling to form the ene-imine C4′–Nα=Cα–Cβ=Cγ structure with the s-*cis* conformation, which is prerequisite for the non-enzymatic symmetry-allowed suprafacial [1,5]-hydrogen shift to complete the catalytic cycle by releasing α-ketobutyrate. The mechanism based on the X-ray snapshots is consistent with the reactivity of MGL toward methionine analogues. The generality of such a mechanism involving non-enzymatic concerted reaction in other PLP enzymes is discussed.

## Introduction

Many enzymes involved in amino acid and amine metabolism bind pyridoxal 5′-phosphate (PLP) as a cofactor and catalyze a wide range of biologically important reactions such as transamination, racemization, deamination, decarboxylation, isomerization, and β- and γ-elimination/substitution^1^. l-Methionine γ-lyase (MGL: EC 4.4.1.11), a PLP-dependent enzyme involved in the transsulfuration pathway, catalyzes the conversion of l-methionine to α-ketobutyric acid through the elimination of methanethiol from l-methionine. The enzyme also accepts l-cysteine, homocysteine, and their analogues as substrates, and catalyzes γ- and β-elimination/substitution^2^. In protozoan parasites and periodontal bacteria, MGL plays essential roles in energy metabolism, methionine homeostasis, isoleucine biosynthesis, and formation of a sulfide storage molecule, *S*-methylcysteine^2–4^. Moreover, MGL from the bacterium *Micromonospora echinospora* is involved in the biosynthesis of antitumor antibiotics^5^. MGL is important to the survival of parasitic protozoa and periodontal bacteria, but is absent in mammals; consequently, MGL is a promising target for chemotherapeutics^2, 6^ and for treating cancers by the introduction of recombinant MGL protein to cause methionine depletion^7^.

The cofactor PLP plays a major role in catalysis by MGL, including transamination and γ-elimination^8, 9^, yet there is little crystallographic data to provide insights into the catalytic mechanism of the enzyme from the standpoint of organic chemistry. It is therefore essential to obtain a comprehensive set of intermediate structures to describe the reaction pathway catalyzed by MGL.

In this manuscript, we suggest a possible catalytic mechanism for MGL from the protozoan parasite *Entamoeba histolytica* (EhMGL1)^10, 11^ based on X-ray snapshots of enzyme-bound reaction intermediates. The main features of the mechanism are that it involves an E1cB mechanism in the γ-elimination step and a non-enzymatic symmetry-allowed suprafacial [1,5]-hydrogen shift at the final stage of the reaction, and this hydrogen shift is supported by a sophisticated symbiosis between PLP and several catalytic residues.

## Results

### Structure determination

**Figure 1:**
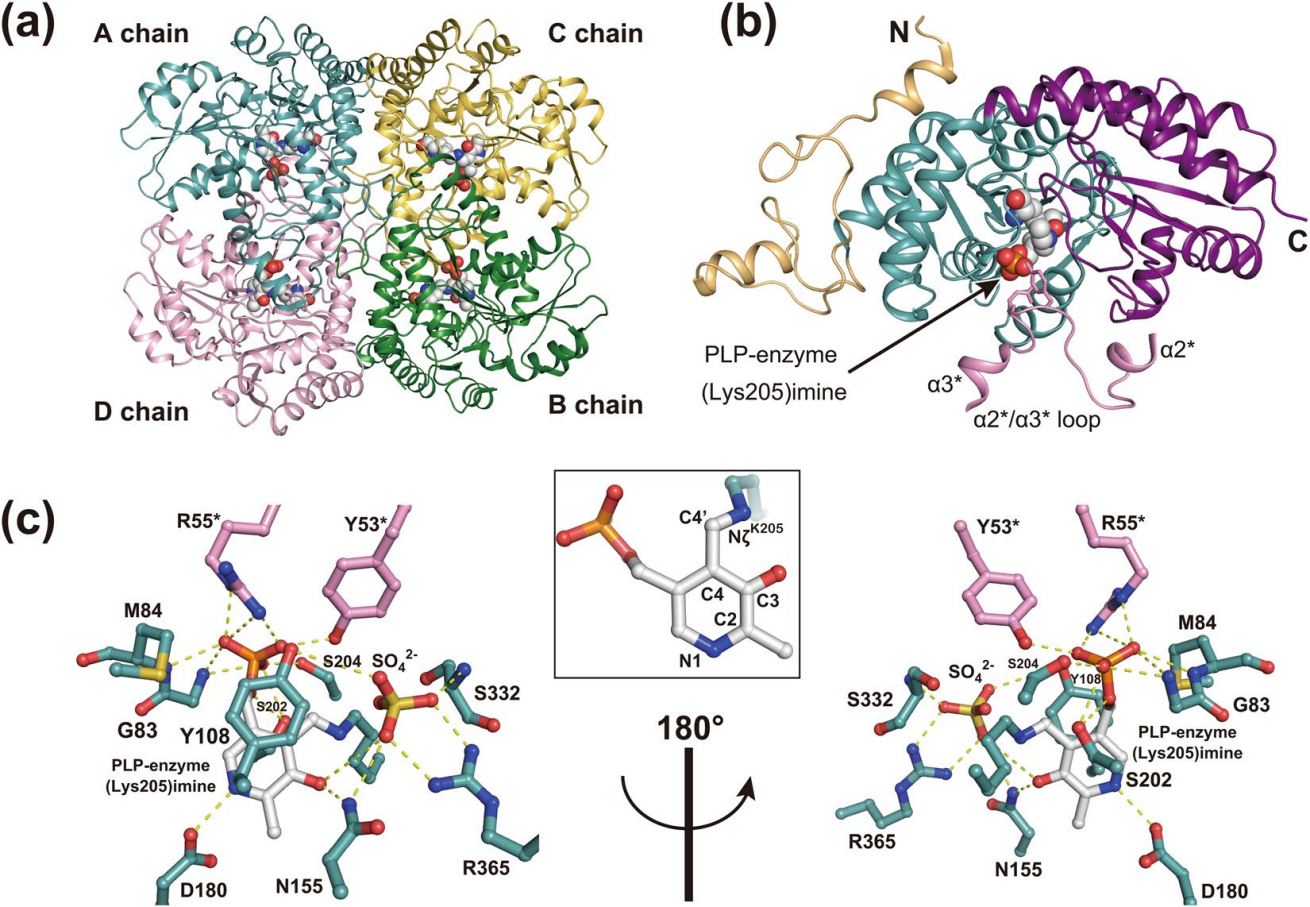
Structure of substrate-free EhMGL1. (**a**) Cartoon representation of the EhMGL1 homotetramer structure. A, B, C and D chains are colored in teal, deep green, yellow and pink, respectively. Two catalytic dimers are formed by A–D and B–C pairs. PLP-enzyme (Lys205) imine bound to each chain is shown as spheres with the color code C (white), N (blue), O (red) and P (orange). (**b**) Structure of the A chain. The N-terminal, PLP-binding and C-terminal domains are colored in light orange, teal and purple, respectively. The bound PLP-enzyme (Lys205) imine is shown as in (**a)**. The α2^*^/α3^*^ loop from the D chain is represented as a pink cartoon and contributes to the formation of the active site of the A chain. (**c**) Stick representation of the active site of the A chain. The color code of PLP-enzyme (Lys205) imine is the same as in (**a**). Residues from the PLP-binding domain are colored in teal, and residues from the α2^*^/α3^*^ loop of the D chain are colored in pink. Dashed lines indicate hydrogen bonds. Numbering of the nitrogen and carbon atoms of PLP is shown in the inset. The images were generated using *PyMOL* ^39^.

The crystal structure of substrate-free EhMGL1 was solved by molecular replacement using the structure of the *Psuedomonas putida* MGL protein (PDB entry 2o7c) as a search model and refined at 1.97 Å resolution (PDB entry 3acz; Supplementary Table S1). Consistent with other MGL proteins of known structure (Supplementary Fig. S1)^5, 12, 13^, EhMGL1 is a homotetrameric enzyme composed of two catalytic dimers (an A–D chain dimer and a B–C chain dimer, Fig. 1a). Each chain comprises an N-terminal domain, a PLP-binding domain, and a C-terminal domain (Fig. 1b) and the active site binds a PLP cofactor that forms a Schiff base with the ε-amino group of Lys205 [PLP-enzyme (Lys205) imine; Fig. 1c]. Six crystal structures with various soaking times in a cryoprotectant solution containing methionine were determined at 2.0–2.6 Å resolution (PDB entries: 3aej, 3aem, 3aen, 3ael, 3aeo and 3aep; Supplementary Table S1). The 28 active sites in the seven structures could be classified into eight stages of catalysis: substrate-free, Michaelis complexes, and intermediates **1a**, **1b**, **2**, **3**, **4a** and **4b**. These assignments are listed in Supplementary Table S2. The overall structure of EhMGL1 is described in Supplementary Results.

### Active sites of substrate-free EhMGL1 and of the Michaelis complex

**Figure 2:**
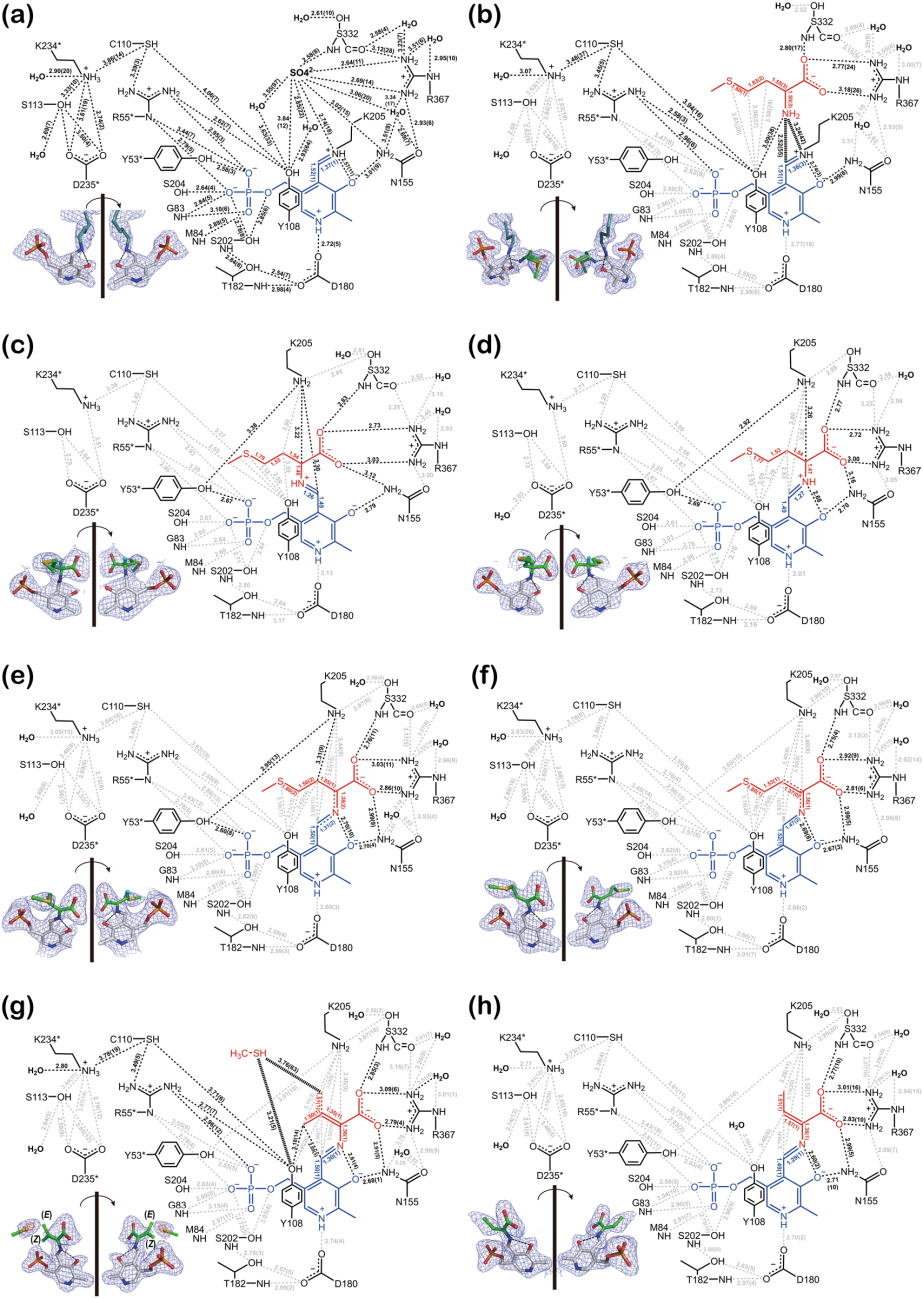
Schematic drawings of the active sites with interatomic distances. (**a**) substrate-free form, (**b**) Michaelis complex, (**c**) intermediate **1a**, (**d**) intermediate **1b**, (**e**) intermediate **2**, (**f**) intermediate **3**, (**g**) intermediate **4a** and an eliminated methanethiol, and (**h**) intermediate **4b**. PLP and substrate moieties of the bound Schiff base are colored in blue and red, respectively. Double configurations about the Cβ–Cγ bond observed in intermediate **4a** are indicated as red solid (*Z* isomer) and dashed (*E* isomer) lines. All interactions in (**a**) and the important interactions in catalysis in (**b**–**h**) are indicated as black dotted lines. For intermediates with multiple determined structures, average interatomic distances (Å) are shown with their standard deviations in parentheses. The structure of each intermediate is shown in inset. The Hα atoms of intermediate **1a** and **1b** and the Hβ atom of intermediate **2** are shown as cyan-colored stick at their calculated positions. Electron densities contoured at 1.0σ and 2.0σ levels are shown as blue and purple cages, respectively. Dashed line indicates a hydrogen bond.

PLP-enzyme (Lys205) imine bound to the active sites of the substrate-free chains is covered by a long α2^*^/α3^*^ loop (Figs 1b,c and 2a; asterisk denotes the adjacent chain of the catalytic dimer) and accepts hydrogen bonds from amino acid residues conserved across amino acid sequences of related PLP enzymes (Supplementary Fig. S1).

The PLP pyridine ring protonated at the N1 atom can form an ion pair with the COO^−^ group of Asp180, and the average distance between the ion pair atoms is 2.72 Å (pertinent bond lengths, including standard deviations, are provided in Fig. 2a). There are potential hydrogen bonds between the PLP pyridine C3 position (O3) and Asn155 (3.01 Å), and between the PLP phosphate group and several residues in the PLP-binding domain (Gly83, Met84, Ser202 and Ser204) and the α2^*^/α3^*^ loop (Tyr53^*^ and Arg55^*^). These hydrogen bonds are observed in all structures determined in this study (Fig. 2a–h). A sulfate ion from the crystallization reagent interacts with Asn155, Lys205, Ser332 and Arg367, and probably promotes crystallization by stabilizing the active site structure of the substrate-free EhMGL1.

In the Michaelis complex (Fig. 2b) the α-carboxyl group of the bound methionine replaces the sulfate ion and accepts hydrogen bonds from the conserved Ser332 and Arg367, and these hydrogen bonds are probably important for binding the methionine molecule to the active site. The methionine α-amino group is in close contact with the C4′=Nζ double bond of PLP-enzyme (Lys205) imine; the distance between the methionine α-amino group and the C4′ and Nζ atoms is 2.52 and 3.24 Å, respectively. The methionine α-amino group forms a hydrogen bond with the hydroxyl group of Tyr108. This hydroxyl group is involved in two hydrogen-bond networks, Tyr108 ··· Arg55*··· PLP PO_4_^2−^ and Tyr108 ··· Cys110 ··· Lys234*··· OH_2_, that respectively connect the methionine α-amino group to the PLP phosphate group and an outside water molecule. Note that the overlap of the *p* orbitals between the C4′=Nζ double bond and the pyridine ring of PLP-enzyme (Lys205) imine is poor, as shown by the C3–C4–C4′–Nζ dihedral angles calculated for the substrate-free enzyme (30.5°) and the Michaelis complex (52.4°) (Supplementary Table S3).

### Intermediates 1a and 1b: conformers of PLP-methionine imine

PLP-methionine imine, a Schiff base formed between PLP and methionine, is bound to the active sites of the 3aej-A, -B and -D chains (Supplementary Table S2) as either intermediate **1a** (Fig. 2c) or **1b** (Fig. 2d). The C3–C4–C4′=Nα dihedral angles of intermediates **1a** (−101.8°) and **1b** (−9.6°) (Supplementary Table S3) show that the pyridine ring and the plane described by the C4′, Nα and Cα atoms intersect approximately at right angles in intermediate **1a**, whereas the three atoms lie almost on the same plane as the pyridine ring in intermediate **1b**. The coplanar conformation of intermediate **1b** results in the formation of an intramolecular hydrogen bond between the Nα and O3 atoms, and the *p* orbitals of the C4′=Nα double bond overlap with those of the pyridine ring. Both the hydrogen bond and the *p* orbital overlap make intermediate **1b** more stable than intermediate **1a**. Since the Nα atom of intermediate **1a** occupies approximately the same position as the methionine Nα atom of the Michaelis complex, intermediate **1a** would be produced first and then spontaneously converted into the energetically more favorable **1b** by a rotation of 92° about the C4–C4′ bond. Since this rotation is accompanied by a rotation of 90° about the Nα–Cα bond in the opposite direction (Supplementary Table S3), the hydrogen bonds of the methionine α-carboxyl group with Asn155, Ser332 and Arg367 observed in the Michaelis complex and intermediate **1a** are preserved in intermediate **1b** and other intermediates (Fig. 2b–h).

### Intermediates 2 and 3: PMP-α-ketomethionine imine and PMP-α,β-dehydromethionine imine

The C4–C4′ bond length of intermediate **2** (1.50 Å; Fig. 2e) agrees with a typical single bond length between aliphatic and aromatic carbon atoms (1.50 Å), and unlike intermediates **1a** and **1b**, the lengths of the C4′–Nα (1.31 Å) and Nα–Cα (1.38 Å) bonds are close to the average of a typical C–N single bond (1.47 Å) and a C=N double bond (1.26 Å). In addition, the planar disposition of the pyridine ring and the C4′, Nα and Cα atoms observed in intermediate **1b** extends to the Cβ atom, as shown by the C3–C4–C4′–Nα (−11.6°), C4–C4′–Nα–Cα (−176.4°) and C4′–Nα–Cα–Cβ (3.9°) dihedral angles (Supplementary Table S3). These observations indicate that intermediate **2** corresponds to PMP-α-ketomethionine imine possessing a conjugated system extending from the pyridine ring to the *p* orbital of the Cα atom.

On the other hand, the C4′ atom of intermediate **3** (Fig. 2f) is not involved in a conjugated system because of the single bond nature of the C4–C4′ (1.52 Å) and C4′–Nα (1.47 Å) bonds. In contrast, the partial double-bond nature of the Nα–Cα (1.36 Å) and Cα–Cβ (1.37 Å) bonds (Fig. 2f) suggests that the structure of intermediate **3** is represented by a resonance hybrid of the enamine form and the charge-separated ylide form of PMP-α,β-dehydromethionine imine resulting from the migration of the β-hydrogen atom of intermediate **2** to the C4′ atom. Due to this proton migration, two conjugated systems of the pyridine ring and the enamine Nα–Cα–Cβ group are distinctly separated by a gap at the saturated C4′ atom, making the existence of a putative quinonoid structure for intermediate **3** unlikely. These resonance structures are illustrated in Supplementary Figure S2.

### Intermediate 4: PLP-α,β-butenoic acid imine

Intermediates **4a** and **4b** lack the γ-methylthio group, as shown by the |2*F*_o_-*F*_c_| electron density maps, and their structures are refined as PLP-α,β-butenoic acid imine with the s-*cis* geometry about the Cα=Cβ and C4′=Nα double bonds (Fig. 2g,h and Supplementary Table S3). In addition, the |2*F*_o_-*F*_c_| maps suggest that intermediate **4a** adopts double *E* and *Z* configurations about the Cα=Cβ double bond, in contrast to the *E* configuration of intermediate **4b**. It is noteworthy that residual electron density, probably representing the eliminated methanethiol molecule, is observed near the Cγ atom of the *E* isomer of intermediate **4a** (Fig. 2g). The average *B*-factor of the methanethiol molecule (63 Å^2^) calculated for the refined model is about twice that of the cofactor and ligand (Supplementary Table S1).

## Discussion

**Figure 3:**
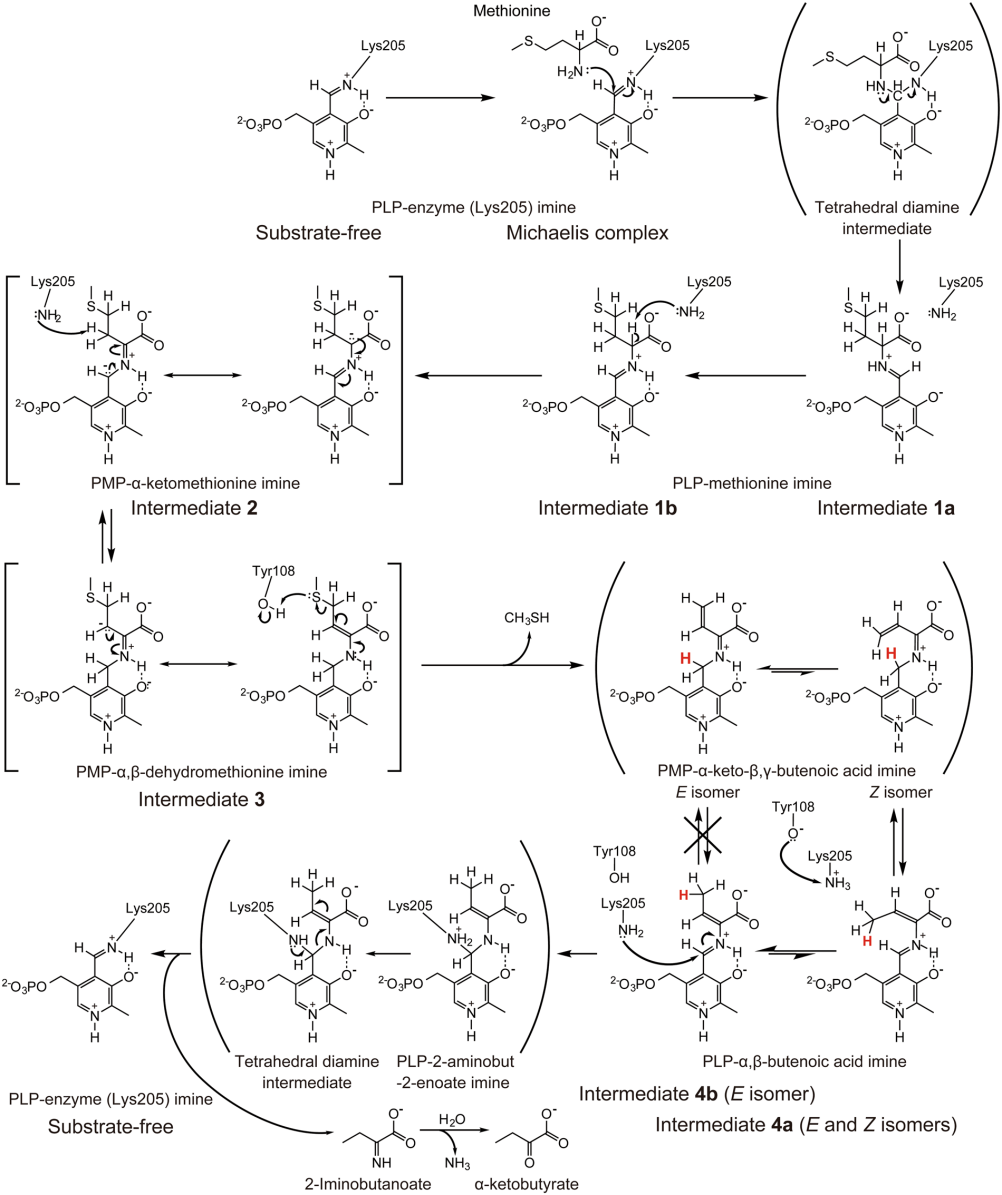
Proposed catalytic reaction mechanism of MGL. Intermediates in parentheses are transient and intermediates in brackets are resonance hybrids. The hydrogen atom involved in suprafacial [1,5]-sigmatropy is shown as red. PLP and PMP denote pyridoxal 5′-phosphate and pyridoxamine 5′-phosphate, respectively.

A series of crystal structures of EhMGL1, including the substrate-free form, Michaelis complex, and complexes with the reaction intermediates **1a**, **1b**, **2**, **3**, **4a** and **4b**, were determined. Based on the reasonable assumption that these intermediates were produced in the crystals by catalysis of crystalline EhMGL1 from the Michaelis complex, and thus represent snapshots of the γ-elimination reaction, we propose here the catalytic mechanism for this enzyme (Fig. 3).

### Step 1: Conversion of the Michaelis complex to PLP-methionine imine

It is conventionally assumed that at the initial stage of catalysis by MGL enzymes, the α-amino group of the methionine molecule in the Michaelis complex substitutes for the ε-amino group of PLP-enzyme (Lys205) imine *via* the tetrahedral diamine intermediate to give PLP-methionine imine^1^. Although the tetrahedral intermediate was not observed in the obtained crystals, the structures of the Michaelis complex and intermediates **1a** and **1b** (Fig. 2b–d) clearly support the conventional mechanism.

When a methionine molecule binds to the active site of EhMGL1, the hydroxyl group of Tyr108 acts as a general base catalyst to remove a proton from the α-NH_3_^+^ group of bound methionine molecule. In this reaction, it appears that the basicity of the Tyr108 hydroxyl group is enhanced by the hydrogen-bond network Tyr108 ··· Arg55*··· PLP PO_4_^2−^, and the removed proton is relayed to an outside water molecule *via* the hydrogen-bond network Tyr108 ··· Cys110 ··· Lys234*··· H_2_O. The hydrogen-bond networks Tyr108 ··· Arg55*··· PLP PO_4_^2−^ and Cys110 ··· Arg55*··· PLP PO_4_^2−^ appear to couple the acid-base equilibrium of the Tyr108 hydroxyl group with that of the Cys110 thiol group to promote the proton relay. The importance of the thiol group is demonstrated by the finding that the specific activities of the C110S and C110G mutant enzymes are 77% and 40%, respectively, that of the wild type enzyme^10^.

Next, nucleophilic attack of the α-amino group of the methionine on the C4′ carbon of PLP-enzyme (Lys205) imine produces intermediate **1a** *via* the transient tetrahedral diamine intermediate, and this intermediate spontaneously changes conformation to yield a more stable intermediate **1b**. This nucleophilic attack appears to be promoted by the enhanced basicity of the α-amino group of the methionine and the positive charge on the C4′=Nζ double bond of PLP-enzyme (Lys205) imine. The former is achieved by the hydrogen-bond network Met α-NH_2_ ··· Tyr108 ··· Arg55*··· PLP PO_4_^2−^, and the latter, as indicated by the C3–C4–C4′=Nζ dihedral angle (52.4°, Supplementary Table S3), by the lack of the overlap between *p*-orbitals of the C4′=Nζ double bond and the PLP pyridine ring. This lack of *p* orbital overlap prevents the positive charge on the C4′=Nζ bond from delocalizing into the PLP pyridine ring.

### Step 2: Formation of PMP-α-ketomethionine imine

Intermediate **2** is a resonance hybrid of two ylide forms, PLP-methionine imine carbanion and PMP-α-ketomethionine imine carbanion (Figs 2e and S2), and is generated by the removal of a proton from the allylic Cα atom of intermediate **1b**. This reaction appears to be catalyzed by the ε-amino group of Lys205 released from PLP-enzyme (Lys205) imine in **Step 1**: it is essentially the sole functional group located close to the calculated position of the α-hydrogen atom in intermediate **1b** (with a distance of 2.36 Å), and its basicity is enhanced by the hydrogen-bond network Lys205 ··· Tyr53*··· PLP PO_4_^2−^ (Fig. 2d). In addition, the Cα–H bond of intermediate **1b** is approximately perpendicular to the plane containing the pyridine ring and the C4′, Nα and Cα atoms (Fig. 2d). This orientation is ideal for the removal of the Cα proton as a consequence of the overlap between the Cα–H bond and the *p*-orbitals of the C4′=Nα bond.

For the base-induced abstraction of the Cα proton from intermediate **1b**, the formation of a pyridoxal *p*-quinoid, often referred to as a quinonoid, has been assumed to represent intermediate **2** in which a conjugated system could stabilize the negative charge on the Cα atom by a resonance effect^14, 15^. However, the single-bond nature of the C4′–C4 bond and the partial double-bond nature of the Cα–Nα and Nα–C4′ bonds of intermediate **2** (Fig. 2e) are not consistent with the structure of a quinonoid. In addition, it is difficult to obtain crucial evidence about the existence of a quinonoid by the measurement of UV/visible spectra, because the absorption of a quinonoid appears at various wavelengths such as 480 nm (threonine synthase)^15^, 500 nm (tyrosine phenol lyase)^16^, and 550 nm (MGL)^17^, which can also vary as the overlap of absorptions arising from a complex mixture of intermediates depicted by a resonance hybrid and in equilibrium between two or more isomers. Even without a resonance effect due to the quinonoid structure, positive charge of the pyridinium group may help stabilize the negative charge through its inductive electron-withdrawing property, as well as the share of a negative charge with the Cα and C4′ atoms. This view is consistent with the report that some PLP dependent enzymes require the electrophilicity of a protonated pyridine ring^18^.

### Step 3: Proton migration to PMP-α,β-dehydromethionine imine and elimination of methanethiol

The PMP-α-keto imine structure of intermediate **2** is a resonance hybrid of two ylide forms (Fig. 3). Because the CH_3_S- group is a poor leaving group, the subsequent γ-elimination should occur by an E1cB mechanism that requires the formation of a carbanion at the Cβ position to expel the CH_3_S- group. However, a strong base appears to be unnecessary to remove the Cβ-H of intermediate **2**: the Nα^+^=Cα–Cβ–H moiety in intermediate **2** is the conjugate acid of the base Nα^+^=Cα–Cβ^−^ in intermediate **3**, and the resulting carbanion form of intermediate **3** is stabilized by resonance. If necessary, a proton can transfer from the ε-amino group of Lys205 located close to the Cβ carbon atom (Fig. 2e), as illustrated in Supplementary Figure S2.

Once the conditions required for the E1cB mechanism are satisfied, the reaction can proceed almost regardless of the geometry of intermediate **3**. In fact, the Hβ–Cβ–Cγ–Sδ dihedral angles of six chains of intermediate **2** range from 111° to 165°, and this range significantly deviates from the anti periplanar geometry (180°) required for the reaction to proceed by an E2 mechanism. In the E1cB mechanism, the rate-determining step should be the breakage of the Xδ–Cγ bond, and the relative rate should follow the increasing order of leaving group ability: CH_3_O^−^ < CH_3_S^−^ < CH_3_Se^− ^^19^. The *k*_cat_ values measured for the wild type enzyme significantly depend on the variation of the leaving Cε–Xδ groups, such that 0.55 s^−1^ (CH_3_Se^−^) < 1.19 s^−1^ (CH_3_O^−^) < 1.82 s^−1^ (CH_3_S^−^), as shown in Table 1. It is notable that the CH_3_Se group, which has the highest leaving group ability, was removed most slowly among these three groups. Such inconsistency between leaving-group ability and reactivity suggests that cleavage of the Xδ–Cγ bond requires the X-atom to be protonated. Because a poor leaving group can be a strong base, the CH_3_O oxygen atom is protonated preferentially amongst O, S and Se atoms, thus enhancing the ability of the group to depart from the Cγ carbon atom. In addition, substituting the S-CH_3_ group with the S-CF_3_ group resulted in lowering the *k*_cat_ value by about 50% **(**Table 1 **)**, probably due to the reduced basicity of the S atom caused by the strong electron-withdrawing effect of the CF_3_ group. The OH group of Tyr108 most likely acts as an acid to protonate the leaving group at the closest distance of 3.18 Å to the Sγ atom (Fig. 2f). The importance of the OH group of Tyr108 is supported by the finding that the Y108F mutant of EhMGL1 is about 15-times less active than the wild type enzyme in terms of the *k*_cat_ value toward methionine (Table 1). Note that the OH group of Tyr108 could act as an acid to protonate the X atom (X=O, S, or Se) of intermediate **3** in **Step 3** and that its conjugate base (phenolate O^−^) is capable of deprotonating the ε-NH_3_^+^ group of Lys205, which is bound to abstract the Cα-proton of methionine in **Step 2** and to facilitate the release of 2-iminobutanoate from intermediate **4b** in **Step 5** (Fig. 3). It is therefore difficult to interpret the rate-enhancing effect of Tyr108 without allowing for the basicity of its anionic form. That is, the *k*_cat_ value of EhMGL1 catalysis consisting of multiple chemical processes may not be determined solely by the rate at a certain stage of the reaction. The more impressive reduction of *k*_cat_ by 910 fold has been found for the Y114F mutant of *P*. *putida* MGL^20^. Since methanethiolate is a potent nucleophile, protonation of its S atom could prevent backward Michael addition.

**Table 1:** Kinetic parameters toward methionine and methionine analogues.

Substrate l-X-CH_2_CH(NH_3_^+^)CO_2_^−^ X=	*K*_M_ (mM)	*k*_cat_ (s^−1^)	*k*_cat_/*K*_M_ (mM^−1^s^−1^)
**Wild type**			
CH_3_S*	0.61 ± 0.06	1.82 ± 0.11	2.99 ± 0.34
CF_3_S*	0.10 ± 0.004	0.81 ± 0.08	8.02 ± 0.83
CH_3_O	2.50 ± 0.24	1.04 ± 0.35	0.42 ± 0.15
CH_3_Se	0.41 ± 0.07	0.55 ± 0.10	1.33 ± 0.34
**Y108F mutant**			
CH_3_S*	1.46 ± 0.12	0.12 ± 0.01	0.08 ± 0.01
CF_3_S*	0.57 ± 0.02	2.22 ± 0.08	3.88 ± 0.18
CH_3_O	4.60 ± 0.37	0.10 ± 0.01	0.02 ± 0.002
CH_3_Se	0.74 ± 0.10	0.06 ± 0.004	0.08 ± 0.01

Values are means ± S.D. (n = 5 or 6).

*These values are referred from our previous study^10^.

### Step 4: Suprafacial [1,5]-hydrogen sigmatropy leading to PLP-α,β-butenoic acid imine

The elimination of methanethiol from intermediate **3** gives PMP-α-keto-β,γ-butenoic acid imine, containing the conjugated C4′–Nα=Cα–Cβ=Cγ structure (Fig. 3). However, the relevant intermediate contained C4′=Nα–Cα=Cβ–Cγ, corresponding to PLP-α,β-butenoic acid imine (Fig. 2g and h). This suggests that PMP-α-keto-β,γ-butenoic acid imine rapidly undergoes migration of a hydrogen atom from the C4′ atom to the Cγ atom, accompanied by the oxidation of PMP-imine to PLP-imine. The refined crystal structures suggest the existence of the *E* and *Z* isomers in intermediate **4a** and the *E* isomer in intermediate **4b** with respect to the configuration about the Cα=Cβ double bond, as described in the **Results** section. Note that residual electron density of methanethiol eliminated from intermediate **3** appeared very close to that of the γ-methyl group of intermediate **4a** (*E* configuration), implying that the γ-methyl group of intermediate **4a** is oriented away from methanethiol to avoid steric hindrance (Fig. 2g). Assuming that the conformation of the precursor is similar to that of intermediate **4a** in the *Z* configuration, the C4′–Nα=Cα–Cβ=Cγ portion of the precursor would adopt the s-*cis* conformation, in which the distance between the Cγ and C4′ atoms is 2.69 Å (Fig. 2g). This distance is short enough for the direct migration of a hydrogen atom.

**Figure 4:**
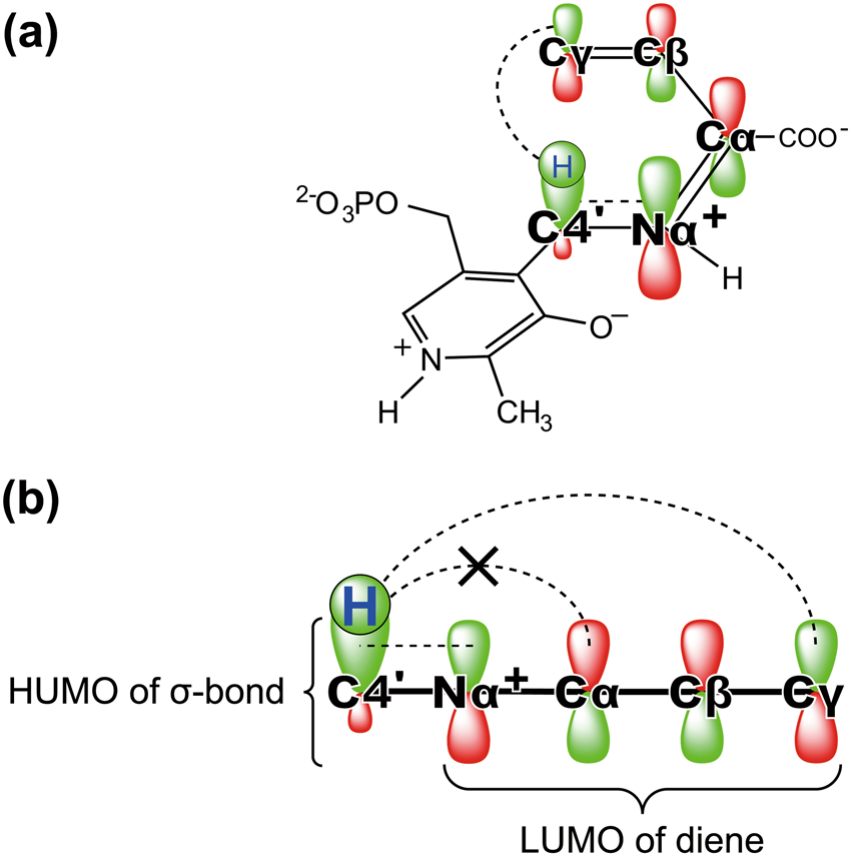
The suprafacial [1,5]-hydrogen shift in a conjugated ene-imine system of PMP-bound butenoate. (**a**) The migration of a hydrogen atom at C4′ to Nα and Cγ is indicated by dashed lines. (**b**) Schematic diagram of the migration of a hydrogen atom. The [1,3]-hydrogen shift from C4′ to Cα is symmetry forbidden.

On these grounds, we propose that this reaction results from suprafacial [1,5]-hydrogen sigmatropy represented by a [σ2 s + π4 s] process in a non-enzymatic pericyclic reaction (Fig. 4a), which is a pericyclic reaction involving a cyclic transition state. In this reaction, scission of the C4′–H bond occurs in concert with formation of the Cγ–H bond according to the strict rules of conservation of orbital symmetry^21^. After the hydrogen transfer, intermediate **4a** with the *Z* configuration isomerizes to the more stable intermediate **4b** with the *E* configuration because of steric hindrance between the C4′ and Cγ atoms, which makes the backward [1,5]-hydrogen shift less probable. This mechanism can also eliminate a difficulty arising when considering a prototropy process because the Cγ carbon of the ene-imine Nα=Cα–Cβ=Cγ system in cystathionine γ-synthase (CGS) does not react with a proton but with a nucleophile during Michael addition^9, 22^.

Concerning the stereochemistry of intermediate **4**, we note that 2-amino-(*Z*)-3-pentenoate acts as an irreversible inhibitor but that the *E* isomer is converted to 2-ketopentanoic acid^17^. This critical difference between the *Z* and *E* isomers can be rationalized by a pericyclic reaction involving a [1,5]-hydrogen shift, which requires the formation of the cyclic transition state, similar to the structure adopted by the *E* isomer in the enzyme. Consequently, the corresponding cyclic intermediate is less likely to arise from the *Z* isomer due to steric interference caused by the δ-methyl group. Unlike an acid- or base-catalyzed *proton transfer*, such as the exchange of a proton between the C4′ and the Cα positions in **Step 1** and **Step 2**, the corresponding [1,3]-*hydrogen shift* by way of a suprafacial transition state is strictly forbidden (Fig. 4b). Therefore, the irreversible inhibition of MGL caused specifically by the 3-(*Z*)-isomer of 2-amino-3-pentenoate represents one of the most crucial phenomena supporting the preference of the symmetry-allowed [1,5]-hydrogen shift over views based on acid-base catalysis. Note that α-vinylglycine has been shown to be converted to α-ketobutyric acid by MGL^23^, CGS^24^, threonine synthase^15^, and even in a non-enzymatic model compound^25^, probably through intermediates similar to the conjugated ene-imine Nα=Cα–Cβ=Cγ system arising from intermediate **3** by elimination. All of these PLP-enzymes could have common features with EhMGL1, and these features provide the substrate with an environment enabling the conjugate system of an intermediate to adopt a cyclic conformation as a transition state during the concerted reaction.

Hydrogen/deuterium (H/D) exchange experiments are often employed to elucidate the reaction mechanism accompanying a shift of proton, hydride, or hydrogen atom. In MGL, a deuterium atom was found incorporated into the γ-position of 2-oxobutanoate from solvent D_2_O, implying an ionic process involving protonation of the γ-carbon atom of PMP-α-keto-β,γ-butenoic acid imine (see Fig. 3 between intermediate **3** and intermediate **4**) might occur^26^. However, this result does not seem to exclude the possibility of [1,5]-hydrogen shift, because at least one of the two hydrogen atoms at the C4′ position of PMP in intermediate **3** has been exchanged with deuterium originated from D_2_O. Obviously, the more elaborated experimental studies are necessary to confirm the present reaction mechanism.

Several possible pericyclic enzymatic reactions include the [3,3]-sigmatropy (the Claisen rearrangement) in chorismate mutase (CM)^27^, the [1,5]-hydrogen shift in isochorismate pyruvate lyase^28, 29^, and the [1,5]-sigmatropic shift of the methyl group in precorrin-8x methyl mutase (CobH)^30^. The reaction mechanisms of CM and CobH have been elucidated on the basis of X-ray crystallographic studies^27, 30^. The most distinguishing feature of a concerted pericyclic reaction is that it does not require catalytic groups to promote the reaction. In agreement with this feature, there were no functional groups such as an acid or a base in the active site of CM. However, the [1,5]-sigmatropy in CobH is enhanced by protonation of the nitrogen atom in the diene moiety^30^. We therefore expect that similar protonation at the Nα-imino nitrogen in the possible precursor of intermediate **4** could facilitate the [1,5]-hydrogen shift.

### Step 5: Regeneration of PLP-bound enzyme with the release of α-ketobutyric acid

This step is essentially the reversal of **Step 1** concerning the conversion of the Michaelis complex to intermediate **1b**. The ε-amino group of Lys205 attacks the imino C4′ carbon atom, replacing the Nα atom of PLP-2-aminobut-2-enoate imine. 2-Aminobut-2-enoate released from the enzyme can isomerize spontaneously to 2-iminobutanoate, which is highly unstable in aqueous solution, followed by its hydrolysis to generate α-ketobutyrate and ammonia^19^.

### Concluding Remarks

In this study, we followed the multi-step reactions of a PLP-dependent enzyme, methionine γ-lyase, by matching the steps with the relevant snapshots of intermediate structures elucidated by X-ray crystallography. The proposed reaction mechanism is summarized in Fig. 3. This mechanism is less complex than anticipated from the multiplicity of reactions involved and assuming only acid-base catalysis by Lys205 and Tyr108. These catalytic residues are responsible for the consecutive migration of a proton associated with the conversion of PLP-methionine imine to the corresponding α,β-ene-imine, which is a direct precursor to the γ-elimination reaction (Fig. 3). The commitment of the enzyme is much less pronounced in **Step 4**; the enzyme serves only to provide the reactant with a space allowing the ene-imine C4′–Nα=Cα–Cβ=Cγ structure to adopt the s-*cis* conformation, which is prerequisite for the symmetry-allowed [1,5]-hydrogen shift. Elucidating the reaction mechanism of an enzyme using conventional methods in enzymology is very difficult, especially when the reaction requires virtually no catalytic group in the case of a concerted reaction. Such a difficulty should be alleviated by careful examination of the stereochemistry associated with progressive changes in the protein-ligand structure. The present catalytic mechanism of MGL can be corroborated by the analysis of X-ray crystallographic snapshots of many other PLP-dependent γ-lyases and γ-synthases. The structures of intermediates, along with the reaction mechanism, could possibly be regarded as seeds for rational design of medicines targeting MGL.

## Methods

### Expression, purification and crystallization of EhMGL1

The recombinant *E*. *histolytica* MGL (EhMGL1), with several silent mutations to prevent fortuitous internal translation, was expressed and purified as described previously^10^. EhMGL1 was crystallized at 277 K using the hanging-drop vapor diffusion method^11^ with minor modifications. A 0.5 μl protein droplet [20 mg/ml in 10 mM HEPES buffer (pH 7.4)] mixed with an equal volume of the reservoir solution [1.8 M ammonium sulfate, 0.1 M cacodylate buffer (pH 6.6), 0.1 M lithium citrate, 100 μM PLP] was equilibrated against 100 μl of the reservoir solution. For data collection at 100 K, a crystal mounted in a nylon loop was briefly soaked in cryo-protectant solution [2.2 M ammonium sulfate, 0.1 M cacodylate buffer (pH 6.2), 0.1 M lithium citrate, 100 μM PLP and 20% (*w*/*v*) glycerol] and frozen in liquid nitrogen. Crystals of EhMGL1 complexed with reaction intermediates were prepared by soaking crystals in the cryo-protectant solution supplemented with 50 mM l-methionine for 30–120 s at 293 K, then freezing in liquid nitrogen. Soaked methionine molecules were converted into reaction intermediates by catalysis by crystalline EhMGL1.

### X-ray diffraction data collection and structure determination

X-ray diffraction experiments were carried out at liquid nitrogen temperature on the following five beamlines: AR-NW12A (ADSC Quantum 210 CCD detector, 3acz and 3aej), BL5A (ADSC Quantum 315 CCD detector, 3ael) and BL17A (ADSC Quantum 270 CCD detector, 3aem and 3aen) at Photon Factory (Tsukuba, Japan); BL41XU (MAR 225 CCD detector, 3aeo) and BL44XU (Bruker DIP-6040 image-plate detector, 3aep) at SPring-8 (Harima, Japan). The applied detector and PDB entries are noted in parentheses. All datasets were integrated and scaled using *HKL2000* ^31^. Statistics of the data collection and processing are shown in Supplementary Table S1. The structure of substrate-free EhMGL1 was determined by molecular replacement using the structure of *P*. *putida* MGL^12^ as a search model using the *Molrep* program^32^, as implemented within the CCP4 package^33^. Structures of methionine-soaked crystals were solved by molecular replacement using the structure of substrate-free EhMGL1 (3acz). For each crystal form, the initial stage of refinement was carried out using the *Refmac5* program^34^ for a model without the Lys205 side chain, PLP cofactor, or methionine molecule. After convergence, the Lys205 side chain, PLP cofactor, and methionine or PLP-methionine imine were modeled into the residual electron densities observed in |2*F*_o_-*F*_c_| and |*F*_o_-*F*_c_| maps. Further refinement using *Refmac5* and manual model correction using *Coot* ^35^ based on the |2*F*_o_-*F*_c_| map were repeated, and six crystal structures of EhMGL1 tetramer were finally determined using the TLS-refinement protocol in *Refmac5* ^36^. Refinement statistics for all models are summarized in Supplementary Table S1.

All crystal structures reported in this study are similar to each other, as indicated by the root-mean-square (r.m.s.) deviations of the Cα positions: 0.08–0.34 Å calculated between protomers in each tetramer, and 0.09–0.24 Å between tetramers. The favored regions, allowed regions, and outliers on the Ramachandran plot (%) calculated by *RAMPAGE* ^37^ are respectively as follows: 98.4, 1.2, 0.3 (3acz); 98.2, 1.5, 0.3, (3aej); 98.1, 1.4, 0.5 (3ael); 97.8, 1.7, 0.5, (3aem); 98.0, 1.4, 0.6 (3aen); 97.5, 1.8, 0.7 (3aeo), 97.8, 1.6, 0.6 (3aep).

### Measurement of enzymological kinetic parameters of MGL

The enzymatic activity of EhMGL1 was measured for the production of α-keto acid^10^. The reaction mixture containing 100 mM phosphate buffer (pH 7.0), 1 mM DTT, 20 μM PLP, and the enzyme (6.25 or 12.5 μg/ml of wild type, or 100 μg/ml of Y108F mutant) was incubated with various concentrations of substrates for 10 min at 37 °C. After terminating the reaction by adding trichloroacetic acid (5%), the released α-ketobutyrate was reacted with 3-methyl-2-benzothiazolinone hydrazone. The amount of the azine derivatives generated was measured by absorbance at 320 nm, which was calibrated with the standard solution of sodium 2-oxobutyrate^38^. The kinetic parameters were estimated using Hanes–Woolf plots.

### Data availability

The crystallographic data collection and refinement statistics, assignment of the intermediates in each chain, dihedral angles, alignment of protein sequences, and resonance structures in intermediate **2** and **3** are available in the online supplementary information.

## Electronic supplementary material

Supplementary Information
