# Supplementary material for: X-Ray snapshots of a pyridoxal enzyme: a catalytic mechanism involving concerted [1,5]-hydrogen sigmatropy in methionine γ-lyase
Source: Sci Rep. 2017 Jul 7;7:4874. doi: 10.1038/s41598-017-05032-6 (PMC5501846; doi:10.1038/s41598-017-05032-6)
Supplement: Supplementary file 1 — Supplementary Information [file 41598_2017_5032_MOESM1_ESM.pdf]

**Supplementary Information**

# X-ray snapshots of a pyridoxal enzyme: a catalytic mechanism involving concerted [1,5]-hydrogen sigmatropy in methionine γ-lyase

Dan Sato[a], Tomoo Shiba[a], Tsuyoshi Karaki[a], Wataru Yamagata[a], Tomoyoshi Nozaki[b], Takashi Nakazawa[c] and Shigeharu Harada[a*]

[a] Graduate School of Science and Technology, Department of Applied Biology, Kyoto Institute of Technology, Sakyo-ku, Kyoto 606-8585, Japan

[b] Department of Parasitology, National Institute of Infectious Diseases, 1-23-1 Toyama, Shinjuku-ku, Tokyo 162-8640, Japan

[c] Department of Chemistry, Nara Women's University, Nara 630-8506, Japan

Supplementary information

**Table of contents** **Page**

Supplementary results | Overall structure of substrate-free EhMGL1 3

Supplementary Table S1 | Data collection and refinement statistics 6

Supplementary Table S2 | Assignment of bound intermediates to the active sites 7

Supplementary Table S3 | Dihedral angles of the intermediates 8

Supplementary Figure S1 | Sequence alignment of EhMGL1 and homologous PLP-dependent enzymes 9

Supplementary Figure S2 | Resonance hybrids observed in intermediates **2** and **3** 11

References 12

**Supplementary results | Overall structure of substrate-free EhMGL1.**

The crystal structure of MGL1 from *Entamoeba histolytica* (EhMGL1) shows that the protein is a homotetramer (A–D chains) composed of two catalytic A-D and B-C dimers (**Fig. 1a**), consistent with other PLP-dependent enzymes with known structures: methionine γ-lyases (MGL) from *Pseudomonas putida* [1] (PDB entry 2o7c), *Trichomonas vaginalis* (1e5f), *Citrobacter freundii* [2] (2rfv), *Micromonospora echinospora* [3] (4u1t), *Clostridium sporogenes* [4] (5dx5), cystathionine β-lyase from *Escherichia coli* [5] (1cl1), cystathionine γ-lyases from yeast [6] (1n8p) and human [7] (2nmp), and cystathionine γ-synthases from *Nicotiana tabacum* [8] (1qgn) and *E. coli* [9] (1cs1). Each protomer consists of three domains: a small N-terminal domain (residues 1–57), the large PLP-binding domain (residues 58–256), and a C-terminal domain (residues 257–389) (**Fig. 1b**). The N-terminal domain includes two α-helices (α1 and α2) connected by a long loop (α1/α2 loop, residues 46–57) and this loop provides most of the contacts with neighboring subunits. The PLP-binding domain includes eight α-helices (α3–α10) and seven β-strands (β1–β7). The strands are arranged in the order β1, β7, β6, β5, β4, β2 and β3 and are oriented ↑, ↓, ↑, ↑, ↑, ↑ and ↑, respectively, and form a predominantly parallel seven-stranded β-sheet. Each strand is connected to the next strand by an α-helix, except for a linker between β6 and β7 (β6/β7 loop, residues 203–215). Five helices (α3, α6, α7, α8

and α10) flank one side of the β-sheet and shield it from solvent, whereas α4, α5 and α9 located on the other side of the β-sheet are involved in the intermolecular interface of the catalytic dimer. The longest α-helix (α10, residues 241–276) is bent at Pro256, and this bend marks the beginning of the C-terminal domain. The C-terminal domain is composed of five α-helices (α10–α14) and five β-strands (β8–β12) that are arranged in the order β8, β9, β12, β11 and β10 and are oriented ↓, ↑, ↓, ↑ and ↑, respectively. One side of the sheet is relatively hydrophobic and is shielded from solvent by α12, α14, and the C-terminal half of α10, whereas the other side comprises all hydrophilic amino acid residues in the five β-strands (Lys282, Asn283, Thr306, Glu310, Thr328, Glu341 and Arg367) that interact with residues from the PLP-binding domain and external water molecules.

In each active site of the EhMGL1 tetramer, a PLP cofactor forms a Schiff base with the ε-amino group of Lys205 [PLP-enzyme (Lys205) imine; **Fig. 1c**]. The Schiff base is covered with a long $\alpha2^*/3^*$ loop (asterisk denotes the adjacent subunit of the catalytic dimer) that shields the base from external water and anchors it to the active site through hydrogen bonds with amino acid residues (**Fig. 1c**). The PLP pyridine N1 atom forms a hydrogen bond with the carboxyl group of Asp180, and this hydrogen bond has an average length of 2.72 Å (5) (pertinent bond lengths, including standard deviations, are shown in parenthesis and were calculated for substrate-free 3acz-A, -B, -C -D and

3aen-C chains). This N1 hydrogen bond probably stabilizes the positive charge at the N1 atom. The hydroxyl group at the pyridine C3 atom receives hydrogen bonds from Lys205 Nζ [2.57 (11) Å] and Asn155 Nδ [3.01 (9) Å]. The phosphate group of PLP forms hydrogen bonds with the Nα atoms of Gly83 and Met84, and the Oγ-atoms of Ser202 and Ser204. The side chains of Tyr53* and Arg55* also interact with the phosphate group through hydrogen bonds. A $SO_4^{2-}$ ion, a component of the crystallization reservoir solution, is bound to the active site and forms hydrogen bonds with Asn155 Nδ, Lys205 Nξ, Ser332 Nα, and the guanidino group of Arg367. These hydrogen bonds appear to stabilize the structure of the substrate-free active site. Amino acid residues involved in interactions with PLP are mostly conserved across amino acid sequences of related PLP enzymes with known structures (**Supplementary Fig. S1**).

**Supplementary Table S1 | Data collection and refinement statistics.**

| PDB entry | 3acz | 3aej | 3ael | 3aem | 3aen | 3aeo | 3aep |
|---|---|---|---|---|---|---|---|
| **Data collection** | | | | | | | |
| Space group | $P\ 2_1$ | $P\ 2_1$ | $P\ 2_1$ | $P\ 2_1$ | $P\ 2_1$ | $P\ 2_1$ | $P\ 2_1$ |
| Cell dimensions | | | | | | | |
| *a*, *b*, *c* (Å) | 99.3, 85.3, 114.6 | 98.7, 85.4, 113.7 | 99.0, 85.2, 114.3 | 99.1, 85.3, 114.3 | 99.2, 85.4, 114.8 | 99.1, 85.7, 115.1 | 99.2, 85.4, 114.7 |
| β (°) | 102.0 | 101.9 | 102.0 | 101.9 | 101.9 | 101.4 | 102.0 |
| Resolution (Å) | 50 – 1.97 (2.04 – 1.97) | 50- 2.59 (2.68 - 2.59) | 50 - 2.0 (2.03 - 2.0) | 50 - 2.2 (2.24 - 2.2) | 50 - 2.1 (2.18 - 2.1) | 50 - 2.15 (2.23 - 2.15) | 50 - 2.28 (2.36 - 2.28) |
| $R_{merge}$ (%) | 5.8 (38.8) | 9.9 (39.3) | 6.6 (36.1) | 8.8 (34.9) | 8.7 (36.8) | 8.9 (39.6) | 9.2 (39.4) |
| $I / \sigma(I)$ | 22.1 (3.5) | 12.4 (3.0) | 26.5 (4.7) | 16.5 (3.6) | 14.1 (2.6) | 13.4 (3.5) | 9.5 (2.0) |
| Completeness (%) | 97.6 (97.1) | 99.9 (99.9) | 100.0 (99.8) | 99.9 (99.4) | 99.4 (95.8) | 100.0 (99.9) | 99.9 (99.4) |
| Redundancy | 3.4 (3.4) | 3.7 (3.6) | 3.7 (3.7) | 3.8 (3.6) | 3.7 (3.1) | 3.8 (3.8) | 3.8 (3.6) |
| **Refinement** | | | | | | | |
| Resolution (Å) | 39.06 - 1.97 (2.02-1.97) | 39.04 - 2.59 (2.65-2.59) | 34.3 - 2.0 (2.05-2.0) | 48.48 - 2.2 (2.25-2.2) | 46.92 - 2.0 (2.05-2.0) | 39.9 - 2.15 (2.20-2.15) | 39.89 - 2.28 (2.34-2.28) |
| No. reflections | 129283 | 57685 | 125424 | 90121 | 117579 | 101969 | 84948 |
| $R_{factor}$ (%) | 15. 6 (19.7) | 20.9 (27.3) | 15.3 (18.9) | 15.1 (18.5) | 16.6 (27.5) | 15.3 (19.3) | 15.6 (24.5) |
| $R_{free}$ (%) | 19.0 (25.0) | 25.6 (33.8) | 18.8 (22.5) | 19.6 (26.2) | 19.8 (28.8) | 19.4 (22.0) | 19.8 (28.1) |
| No. atoms | | | | | | | |
| Protein | 11814 | 11761 | 11764 | 11764 | 11764 | 11830 | 11764 |
| Cofactor and ligand | 60 [a)] | 96 [a)] | 113 | 96 [a)] | 83 [a)] | 96 | 130 |
| Sulfate ion and glycerol | 72 | 32 | 32 | 32 | 37 | 32 | 32 |
| Water | 1005 | 419 | 927 | 890 | 874 | 736 | 694 |
| Average *B*-factors | | | | | | | |
| Protein | 27.72 | 28.54 | 29.77 | 44.03 | 31.80 | 36.67 | 32.03 |
| Cofactor and ligand | 19.76 [a)] | 31.44 [a)] | 27.02 | 27.72 [a)] | 26.36 [a)] | 33.92 | 27.98 |
| Sulfate ion and glycerol | 66.80 | 55.92 | 60.15 | 57.77 | 58.02 | 66.22 | 69.81 |
| Water | 15.11 | 27.28 | 34.48 | 30.69 | 34.64 | 35.57 | 33.34 |
| R.m.s. deviations | | | | | | | |
| Bond lengths (Å) | 0.016 | 0.006 | 0.016 | 0.016 | 0.015 | 0.016 | 0.015 |
| Bond angles (°) | 1.468 | 0.938 | 1.524 | 1.537 | 1.483 | 1.484 | 1.535 |

X-ray diffraction data were collected on one crystal for each structure. Values in parentheses are for highest-resolution shell.

[a)] Lys205 moieties of PLP-enzyme (Lys205) imine are excluded from the calculations.

**Supplementary Table S2 | Assignment of bound intermediates to the active sites.**

| Chain / PDB entry | A chain | B chain | C chain | D chain |
|---|---|---|---|---|
| 3acz | Substrate-free | Substrate-free | Substrate-free | Substrate-free |
| 3aej | Intermediate **1a** | Intermediate **1b** | Michaelis complex | Intermediate **1a** |
| 3ael | Intermediate **4a** | Intermediate **2** | Intermediate **2** | Intermediate **2** |
| 3aem | Intermediate **2** | Intermediate **2** | Michaelis complex | Intermediate **2** |
| 3aen | Michaelis complex | Intermediate **4b** | Substrate - free | Intermediate **4b** |
| 3aeo | Intermediate **3** | Intermediate **3** | Intermediate **3** | Intermediate **3** |
| 3aep | Intermediate **4a** | Intermediate **4b** | Intermediate **4a** | Intermediate **4b** |

**Supplementary Table S3 | Dihedral angles of the intermediates.**

| | C3–C4–C4'–Nζ$^{K205}$ | C3–C4–C4'–N$^{Met}$ | C3–C4–C4'–Nα | C4–C4'–Nα–Cα | C4'–Nα–Cα–Cβ | Nα–Cα–Cβ–Cγ | Cα–Cβ–Cγ–Sδ |
|---|---|---|---|---|---|---|---|
| Substrate-free | 30.5 ± 6.0 | | | | | | |
| Michaelis complex | 52.4 ± 4.5 | -93.5 ± 24.8 | | | | | |
| Intermediate **1a** [a)] | 106.9 | | -101.8 | 178.8 | 128.5 | 118.0 | -161.5 |
| Intermediate **1b** [a)] | 88.9 | | -9.6 | 179.7 | 39.4 | 150.0 | -168.0 |
| Intermediate **2** | 88.0 ± 2.9 | | -11.6 ± 3.9 | -176.4 ± 1.3 | 3.9 ± 2.9 | 155.2 ± 6.3 | -111.6 ± 16.8 |
| Intermediate **3** | 74.2 ± 9.6 | | -6.3 ± 7.2 | -163.5 ± 3.3 | -7.9 ± 9.2 | 178.4 ± 1.2 | -91.1 ± 6.0 |
| Intermediate **4a** [b)] | 88.7 ± 2.1 | | -17.3 ± 1.5 | -177.8 ± 0.5 | (*E*) 1.7 ± 1.6<br>(*Z*) 2.6 ± 1.1 | (*E*) 179.6 ± 0.4<br>(*Z*) -0.5 ± 0.8 | |
| Intermediate **4b** | 88.6 ± 3.4 | | -8.6 ± 4.4 | -177.4 ± 0.9 | 2.4 ± 0.3 | 179.8 ± 0.5 | |

[a)] Standard deviations are not noted due to less than three data.

[b)] Dihedral angles of Nα–Cα–Cβ–Cγ in intermediate **4a** are calculated for *E* and *Z* configurations.

## Supplementary Figure S1

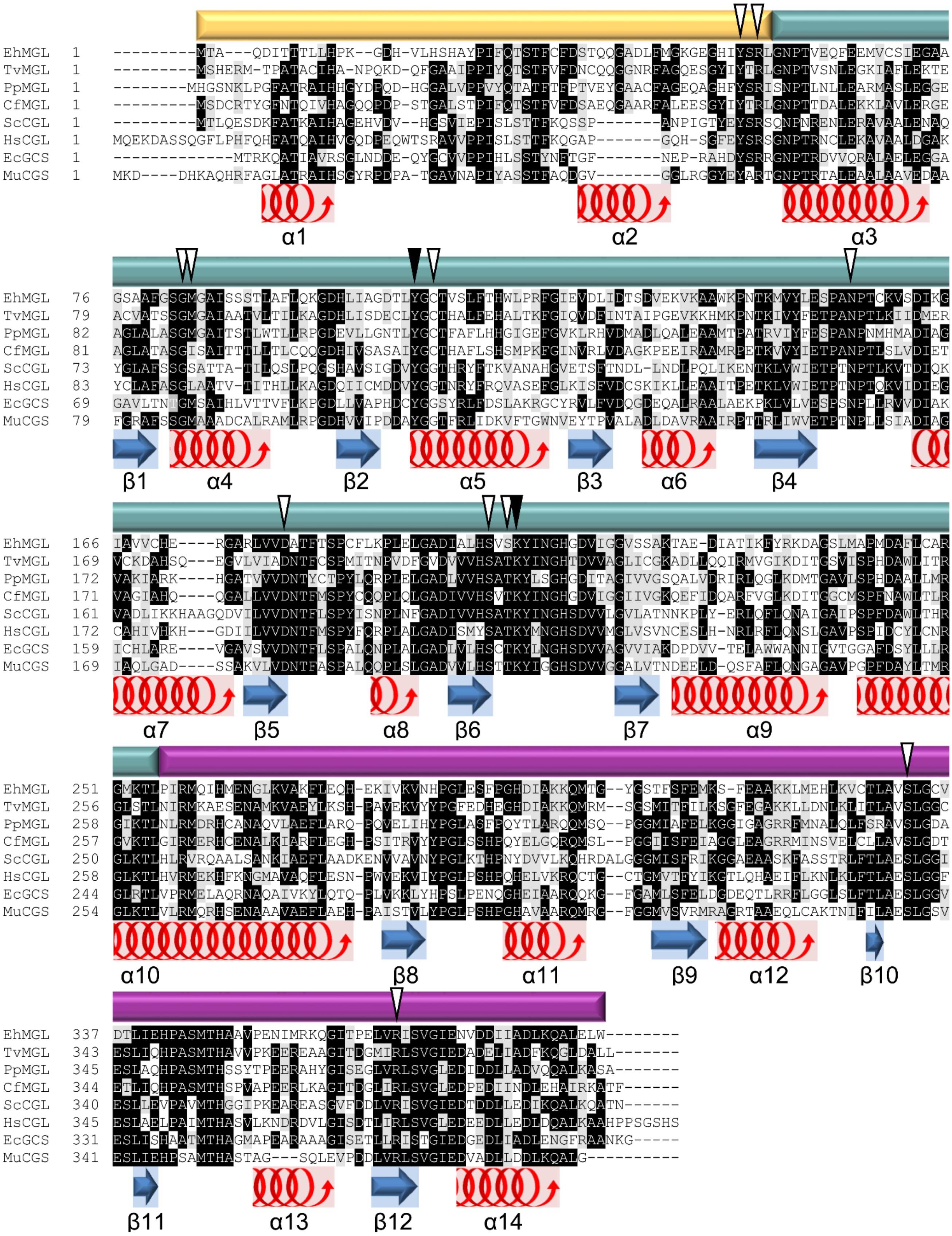

**Supplementary Figure S1 | Sequence alignment of EhMGL1 and homologous PLP-dependent enzymes.** Amino acid sequences of MGLs from *E. histolytica* (PDB entry, 3acz), *T. vaginalis* (1e5f), *P. putida* [1] (2o7c), *C. freundii* [2] (2rfv), cystathionine γ-lyases from yeast [6] (1n8p) and human [7] (2nmp), and cystathionine γ-synthases from *E. coli* [9] (1cs1) and *Mycobacterium ulcerans* Agy99 [10] (3qi6) were aligned using Clustal Omega [11]. Tyr108 and Lys205, which are essential residues for catalysis by EhMGL1, are marked with black triangles. Residues interacting with reaction intermediates are indicated by white triangles. N-terminal, PLP-binding, and C-terminal domains are indicated by light orange, teal, and purple bars, respectively. Regions of α-helices and β-sheets are indicated as red spirals and blue arrows, respectively.

**Supplementary Figure S2**

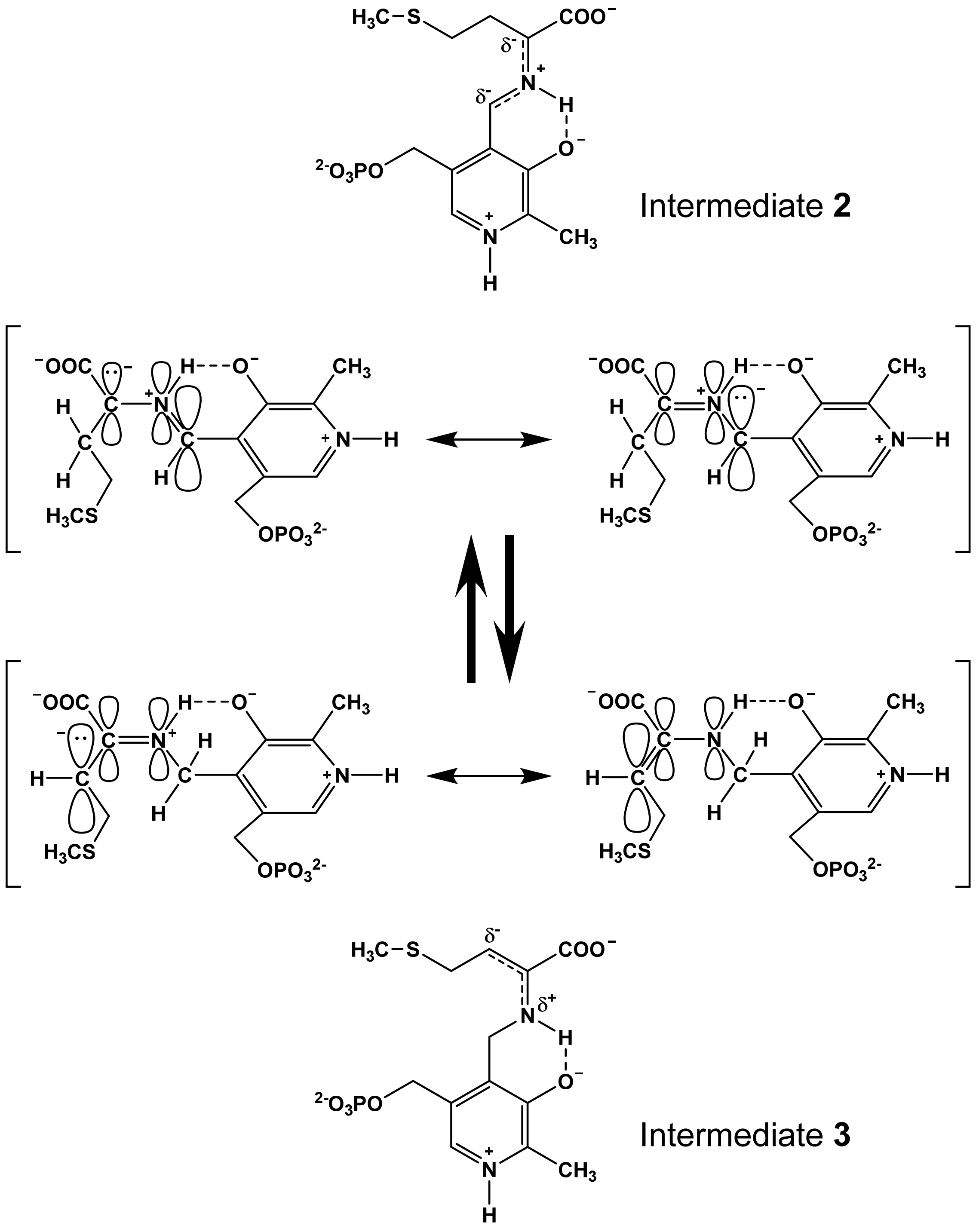


**Supplementary Figure S2 | Resonance hybrids observed in intermediates 2 and 3.**

**References**

1. Kudou, D. *et al.* Structure of the antitumour enzyme L-methionine γ-lyase from *Pseudomonas putida* at 1.8 Å resolution. *J. Biochem. (Tokyo).* **141**, 535-544 (2007).
2. Nikulin, A. *et al.* High-resolution structure of methionine γ-lyase from *Citrobacter freundii*. *Acta Cryst. D.* **64**, 211-218 (2008).
3. Song, H., Xu, R. & Guo, Z. Identification and characterization of a methionine γ-lyase in the calicheamicin biosynthetic cluster of *Micromonospora echinospora*. *ChemBioChem* **16**, 100-109 (2014).
4. Revtovich, S. *et al.* Structure of methionine γ-lyase from *Clostridium sporogenes*. *Acta Cryst. F.* **72**, 65-71 (2016).
5. Clausen, T., Huber, R., Laber, B., Pohlenz, H.D. & Messerschmidt, A. Crystal structure of the pyridoxal-5'-phosphate dependent cystathionine β-lyase from *Escherichia coli* at 1.83 Å. *J. Mol. Biol.* **262**, 202-224 (1996).
6. Messerschmidt, A. *et al.* Determinants of enzymatic specificity in the Cys-Met-metabolism PLP-dependent enzymes family: crystal structure of cystathionine γ-lyase from yeast and intrafamiliar structure comparison. *Biol. Chem.* **384**, 373-386 (2003).
7. Sun, Q. *et al.* Structural basis for the inhibition mechanism of human cystathionine γ-lyase, an enzyme responsible for the production of $H_2S$. *J. Biol. Chem.* **284**, 3076-3085 (2009).

8. Steegborn, C. *et al.* Kinetics and inhibition of recombinant human cystathionine γ-lyase. Toward the rational control of transsulfuration. *J. Biol. Chem.* **274**, 12675-12684 (1999).

9. Clausen, T., Huber, R., Prade, L., Wahl, M.C. & Messerschmidt, A. Crystal structure of *Escherichia coli* cystathionine γ-synthase at 1.5 Å resolution. *EMBO J.* **17**, 6827-6838 (1998).

10. Clifton, M.C. *et al.* Structure of the cystathionine γ-synthase MetB from *Mycobacterium ulcerans*. *Acta Cryst. F.* **67**, 1154-1158 (2011).

11. Sievers, F. *et al.* Fast, scalable generation of high-quality protein multiple sequence alignments using Clustal Omega. *Mol. Syst. Biol.* **7**, 539 (2011).
